# Contemporary treatment of children with critical and near-fatal asthma

**DOI:** 10.5935/0103-507X.20160020

**Published:** 2016

**Authors:** Steven L. Shein, Richard H. Speicher, José Oliva Proença Filho, Benjamin Gaston, Alexandre T. Rotta

**Affiliations:** 1 Division of Pediatric Critical Care Medicine, UH Rainbow Babies & Children's Hospital, Case Western Reserve University School of Medicine - Cleveland, OH, United States.; 2 Division of Pediatric Critical Care Medicine and Neonatology, Hospital e Maternidade Brasil - Santo André (SP), Brazil.; 3 Division of Pediatric Pulmonology, UH Rainbow Babies & Children's Hospital, Case Western Reserve University School of Medicine - Cleveland, OH, United States.

**Keywords:** Asthma, Respiration, artificial, Child

## Abstract

Asthma is the most common chronic illness in childhood. Although the vast majority of children with acute asthma exacerbations do not require critical care, some fail to respond to standard treatment and require escalation of support. Children with critical or near-fatal asthma require close monitoring for deterioration and may require aggressive treatment strategies. This review examines the available evidence supporting therapies for critical and near-fatal asthma and summarizes the contemporary clinical care of these children. Typical treatment includes parenteral corticosteroids and inhaled or intravenous beta-agonist drugs. For children with an inadequate response to standard therapy, inhaled ipratropium bromide, intravenous magnesium sulfate, methylxanthines, helium-oxygen mixtures, and non-invasive mechanical support can be used. Patients with progressive respiratory failure benefit from mechanical ventilation with a strategy that employs large tidal volumes and low ventilator rates to minimize dynamic hyperinflation, barotrauma, and hypotension. Sedatives, analgesics and a neuromuscular blocker are often necessary in the early phase of treatment to facilitate a state of controlled hypoventilation and permissive hypercapnia. Patients who fail to improve with mechanical ventilation may be considered for less common approaches, such as inhaled anesthetics, bronchoscopy, and extracorporeal life support. This contemporary approach has resulted in extremely low mortality rates, even in children requiring mechanical support.

## Abstract (pt)

A asma é a mais comum das doenças da infância. Embora a maioria das crianças com exacerbações agudas de asma não demanda cuidados críticos, algumas delas não respondem ao tratamento padrão e necessitam de cuidados mais intensos. Crianças com asma crítica ou quase fatal precisam de monitoramento estrito quanto à deterioração e podem requerer estratégias terapêuticas agressivas. Esta revisão examinou as evidências disponíveis que dão suporte a terapias para asma crítica e quase fatal, e resumiu o cuidado clínico atual para essas crianças. O tratamento típico inclui uso parenteral de corticosteroides e fármacos beta-agonistas, por via inalatória ou intravenosa. Para crianças com resposta inadequada ao tratamento padrão, pode-se lançar mão do uso inalatório de brometo de ipratrópio ou intravenoso de sulfato de magnésio, metilxantinas e misturas gasosas com hélio, além de suporte ventilatório mecânico não invasivo. Pacientes com insuficiência respiratória progressiva se beneficiam de ventilação mecânica com uma estratégia que emprega grandes volumes correntes e baixas frequências do ventilador, para minimizar a hiperinsuflação dinâmica, o barotrauma e a hipotensão. Sedativos, analgésicos e bloqueadores neuromusculares são frequentemente necessários na fase inicial do tratamento para facilitar um estado de hipoventilação controlada e hipercapnia permissiva. Pacientes que não conseguem melhorar com a ventilação mecânica podem ser considerados para abordagens menos comuns, como inalação de anestésicos, broncoscopia e suporte extracorpóreo à vida. Esta abordagem atual resultou em taxas de mortalidade extremamente baixas, mesmo em crianças com necessidade de suporte mecânico.

## INTRODUCTION

Asthma is the most common chronic illness in childhood, affecting approximately 10% of all children.^(1,2)^ Asthma exacerbations ("attacks") frequently prompt hospitalization, with approximately 150,000 pediatric asthma admissions occurring in the United States annually.^(3)^ Several terms are used to denote severe asthma attacks, including *status asthmaticus, acute severe asthma, critical asthma* and *near-fatal asthma*. Definitions vary among sources, and many consider "status asthmaticus" to be an outdated term.^(4-8)^ For this review, "acute severe asthma" is defined as an asthma attack unresponsive to repeated doses of beta-agonists and requiring hospital admission;^(4)^ "critical asthma" is defined as acute severe asthma necessitating intensive care unit (ICU) admission due to clinical worsening or failure to improve, a need to intensify treatment or escalate support, and a need for continued close monitoring;^(9,10)^ and "near-fatal asthma" is defined as critical asthma with progressive respiratory failure, fatigue, and altered consciousness that requires endotracheal intubation and mechanical ventilation.^(10)^ This review will focus on the management of critical asthma and near-fatal asthma, both of which are increasingly common conditions.^(3,11,12)^ The epidemiology and pathophysiology of asthma have been exhaustively reviewed elsewhere.

## DIAGNOSIS

Critical asthma is a clinical diagnosis. Children often present with dyspnea, tachypnea and wheezing due to severe airway obstruction from inflammation-mediated airway edema, mucus hypersecretion, airway plugging, and bronchospasm. Symptoms often are triggered by either a viral respiratory infection or exposure to an allergen. A prior history of asthma and other risk factors for severe disease are suggestive but not always present (Table 1). In fact, of 260 children with near-fatal asthma at 8 US centers in the Collaborative Pediatric Critical Care Research Network (CPCCRN), 13% had no prior history of asthma, and only 37% of known asthmatics had required hospitalization in the 12 months preceding the episode of near-fatal asthma.^(10)^

**Table 1:** Risk factors for near-fatal asthma

Medical factors
Underuse of controller therapy (e.g., inhaled steroids)
High consumption (> 2 canisters per month) of β-agonist metered-dose inhalers
Previous asthma attack with:
Admission to intensive care unit
Respiratory failure and mechanical ventilation
Seizures or syncope
PaCO_2_ > 45 torr
Psychosocial factors
Denial of or failure to perceive severity of illness
Associated depression or other psychiatric disorder
Noncompliance
Dysfunctional family unit
Ethnic factors
Nonwhite children (black, Hispanic, other)

PaCO_2_ - partial pressure of carbon dioxide.

Diagnostic studies beyond a history and physical examination usually are not required but may be helpful. A chest radiograph typically shows hyperinflated lungs and may also identify pneumothorax, pneumonia, anatomic abnormalities (e.g., vascular rings or a right-sided aortic arch) or foreign bodies. Chest radiography is essential in near-fatal asthma, and we typically obtain a radiograph at admission to the ICU. Routine blood chemistry analysis and blood cell counts generally are not helpful in critical asthma, although they may be indicated in patients at risk for electrolyte imbalances secondary to dehydration or medication effects. If a blood count is obtained, leukocytosis must be interpreted cautiously as it may reflect a demargination response to endogenous or exogenous corticosteroids and not infection. An arterial blood gas analysis is rarely helpful in critical asthma, as the decision to perform endotracheal intubation typically is made based on physical exam findings. We generally restrict arterial blood gas analyses to patients with near-fatal asthma in whom it is used to monitor disease progression and the adequacy of mechanical ventilation support. However, a blood gas analysis may be the only means to diagnose significant hypercarbia in critical asthma patients who have altered mentation from neurologic co-morbidities or static encephalopathy.

## TREATMENT - PRE-INTENSIVE CARE UNIT

Most patients with critical asthma are admitted to the ICU due to an inadequate response to typical therapy in the Emergency Department: systemic corticosteroids, a 1 to 3 hour period of frequent (e.g., every 20 minutes) or continuous albuterol, and 2-3 doses of nebulized ipratropium bromide.^(13)^ Intravenous magnesium administration in the Emergency Department may reduce the rates of hospitalization.^(14,15)^ Intravenous fluids should be provided for dehydration, oxygen for hypoxemia, and antibiotics if there is evidence of a concomitant bacterial infection. Criteria for admission to the ICU vary between centers but may include the need for frequent (e.g., every 1 hour) or continuous albuterol, the need for positive pressure ventilation, severe hypoxemia, or high likelihood of progression to respiratory failure.

## GENERAL INTENSIVE CARE UNIT CARE

Patients with critical asthma represent a heterogeneous group requiring different levels of monitoring and treatment. However, all critical asthma patients warrant continuous monitoring of heart rate, respiratory rate, pulse oximetry (Spo_2_), and noninvasive blood pressure measurements. Arterial and central venous catheters should be placed in patients with near-fatal asthma. Supplemental oxygen should be provided if hypoxemia is present, which is common due to ventilation-perfusion mismatch and intrapulmonary shunts caused by mucus plugging, atelectasis and hyperinflation. β-agonist use may exacerbate hypoxemia by abolishing regional pulmonary hypoxic vasoconstriction and increasing intrapulmonary shunt.^(16,17)^ We generally aim to maintain arterial oxygen saturations greater than 92% in patients admitted to our ICU, although lower thresholds (88 - 90%) may be tolerated as long as systemic oxygen delivery is adequate. Dehydration is common due to decreased oral fluid intake and increased insensible water losses, but fluid resuscitation should be judicious to avoid volume overload and minimize the chance of clinically significant pulmonary edema. Patients should remain NPO and on isotonic intravenous (IV) fluids until a sustained improvement in respiratory status allows for the safe initiation of enteral nutrition. In patients with near-fatal asthma, additional intravenous fluid usually is required to maintain adequate preload during the initiation of positive pressure ventilation. Antimicrobials are not a standard therapy for critical asthma. Antibiotics should be administered if bacterial pneumonia is highly suspected, and early antiviral therapy should be provided for patients infected with influenza virus.

## CORTICOSTEROIDS

Corticosteroids play a central role in the treatment of patients with critical and near-fatal asthma, considering that these conditions are predominantly inflammatory in nature. Corticosteroids modulate airway inflammation by a number of mechanisms, including suppression of a wide range of cytokines (e.g., Interleukins-1, -4, -5, -6, -13), adhesion molecules, and inducible enzymes, including NO-synthase and cyclooxygenase-2.^(18)^ In addition, corticosteroids increase the density, affinity and functionality of β-adrenergic receptors in both normal and catecholamine-desensitized conditions, thus increasing the efficacy of co-administered β-adrenergic agents.^(19)^ This mechanism may explain, at least in part, the rapid clinical improvement exhibited by some patients treated with a combination of corticosteroid and β-adrenergic agents. Corticosteroids also decrease airway mucus production, reduce inflammatory cell infiltration and activation, and attenuate capillary permeability.^(20-23)^

In children with critical or near-fatal asthma, corticosteroids should be administered by the IV route. The oral route may be used in selected cases, but inhaled corticosteroids play no role in the treatment of the hospitalized patient.^(24,25)^ The most common agent used in the United States is methylprednisolone because of its wide availability as an IV preparation and minimal mineralocorticoid effects. We typically administer a loading dose of 2mg/kg of methylprednisolone IV, followed by 0.5mg/kg/dose every 6 hours for 5 to 7 days. Longer treatment courses necessitate gradual weaning of the drug to decrease the chances of symptomatic adrenal insufficiency or relapse. Hydrocortisone, an agent with both glucocorticoid and mineralocorticoid activity, can be used as an alternative at doses of 2 to 4mg/kg/dose IV every 6 hours. Short courses of corticosteroids usually are well tolerated without significant adverse effects.^(22)^ However, hypertension, hyperglycemia, mood disorders, and serious viral infections, such as fatal varicella, have been reported in patients with asthma treated with corticosteroids.^(22,26,27)^ Duration of corticosteroid therapy is dictated by the severity of illness and clinical response, but airway inflammation continues long after the clinical symptoms improve. Prophylaxis with an H_2_ blocker or proton pump inhibitor should be considered because of the possibility of steroid-associated gastritis and gastric perforation.^(28)^

## β-AGONISTS

β-agonists, along with systemic corticosteroids, are the mainstay of pharmacotherapy in persons with critical and near-fatal asthma. β-agonists cause bronchodilation via activation of adenylyl cyclase, resulting in increased intracellular cyclic adenosine monophosphate (cAMP) levels. These agents also can increase diaphragmatic contractility, enhance mucociliary clearance, and inhibit bronchospastic mediators from mast cells.^(29)^ Common side effects of β-agonists include hypoxemia, hypokalemia, tremor, nausea and tachycardia. Less common but more severe cardiac side-effects include diastolic hypotension, cardiac dysrhythmias and myocardial ischemia.^(30-33)^

β-agonists are provided by the inhaled or parenteral routes; there is no role for enteral formulations of these agents in critical or near-fatal asthma. Albuterol (salbutamol) is commonly used as the inhaled agent. Upon ICU admission, most patients with critical or near-fatal asthma are treated with continuous albuterol nebulization. Children with critical asthma randomized to continuous albuterol therapy had more rapid clinical improvement and shorter hospitalizations than children treated with intermittent albuterol doses in one small trial.^(34)^ Continuous administration of albuterol was also associated with more efficient allocation of respiratory therapists' time^(34)^ and could offer the added advantage of more hours of uninterrupted sleep to patients who often are already exhausted.^(35)^ The usual dose of continuously administered albuterol ranges between 0.15 and 0.45mg/kg/h, with a maximum dose of 20mg/h. Higher doses of albuterol have been used in patients who are unresponsive to standard treatment, but we do not find this practice particularly helpful.^(36)^ It should be remembered that major components of bronchial obstruction in severe asthma are mucus and airway wall edema, neither of which is responsive to bronchodilators. Continuous levalbuterol, the pure active enantiomer of albuterol, is more expensive (M.L. Biros, PharmD, Rainbow Babies & Children's Hospital, personal communication, 2016) but not more effective than continuous albuterol.^(37)^ Our standard approach is to provide 15mg/h of continuously nebulized albuterol until the respiratory status improves, and then use intermittent nebulized albuterol (2.5mg/dose) with a sequentially decreasing frequency (i.e., q1h to q2 to q3h to q4h).

Parenteral β-agonists are indicated in children in whom inhaled therapy cannot reach the distal airways due to inadequate air movement or intolerance of the inhalational interface. Intravenous albuterol is not available in the United States but is effective.^(38,39)^ Terbutaline is the most commonly used parenteral β-agonist in the United States. Because of its lower β_1_-receptor affinity, subcutaneous administration of terbutaline has largely supplanted the use of epinephrine in persons with severe acute asthma. Subcutaneous terbutaline is used for patients with acute worsening of the respiratory status who lack vascular access and in whom access cannot be easily obtained, typically in the non-ICU setting. The usual subcutaneous terbutaline dose is 0.01mg/kg/dose (maximum 0.25mg) subcutaneously every 20 minutes for up to three doses, as necessary. Terbutaline is more commonly administered in the ICU by IV infusion. The usual range of IV terbutaline dosage is 0.1 to 10µg/kg/min as a continuous infusion.^(30)^ In our clinical experience, however, most patients are started on a dose of 1µg/kg/min,and the dose is titrated to effect, with doses higher than 4µg/kg/min rarely necessary. Patients starting therapy at doses lower than 1µg/kg/min can be given a loading dose of 10µg/kg over 10 minutes to accelerate the onset of action. Retrospective data suggest that IV terbutaline may reduce the need for mechanical ventilation, but definitive prospective evidence of efficacy in critical or near-fatal asthma is lacking.^(40)^ While hypokalemia is rare with typical doses of inhaled β-agonists, serum potassium levels often decrease by 0.5 to 1.0mEq/L with intravenous infusions of β-agonist agents.^(41-43)^ β-agonist-induced hypokalemia is the result of a potassium shift to the intracellular space in the setting of stable total body potassium, so potassium levels normalize quickly after cessation of the β-agonist infusion. This transient hypokalemia rarely is clinically significant and typically does not require aggressive treatment. We routinely add potassium chloride (20 to 40mEq/L) to the maintenance IV fluid solution and reserve bolus administration of potassium chloride (0.5 to 1mEq/kg [maximum 20mEq/dose], PO or IV) for clinically symptomatic patients with serum potassium measurements below 3.0mEq/L.

## ANTICHOLINERGIC AGENTS

Anticholinergic agents produce bronchodilation by inhibition of cholinergic-mediated bronchospasm, likely by decreasing cyclic guanosine monophosphate.^(44)^ Ipratropium bromide is preferred over atropine as it does not cross the blood-brain barrier to cause central anticholinergic adverse effects, and it does not inhibit ciliary beating and mucociliary clearance.^(44)^ However, extrapulmonary effects such as mydriasis and blurred vision have been reported as a result of inadvertent topical ocular absorption of the nebulized drug.^(45,46)^ The combined use of ipratropium bromide (500µg doses) and nebulized albuterol in treating children with asthma who present to the emergency department has proved to be cost effective and reduces the rate of admission to the hospital.^(13,47)^ However, ipratropium bromide does not improve outcomes in children with acute severe asthma cared for on the general wards.^(48,49)^ Considering the high safety profile of inhaled ipratropium bromide and its clear benefit when used in the emergency department, we typically administer ipratropium bromide along with standard therapy for critically ill patients with asthma despite the lack of robust data specific to the pediatric ICU population.

## MAGNESIUM SULFATE

Magnesium is a physiologic calcium antagonist that inhibits calcium uptake and relaxes bronchial smooth muscle. It usually is administered intravenously, as nebulized magnesium has not been shown to shorten length of hospitalization.^(50)^ The indication for IV magnesium sulfate in children with critical or near-fatal asthma is still unclear because of the paucity of randomized controlled trials. Some studies suggest that magnesium sulfate infusions are associated with significant improvements in short-term pulmonary function,^(14,51,52)^ whereas another study failed to show improvement in disease severity or a reduction in hospitalization rates.^(15)^ The usual dose of magnesium sulfate in children with critical or near-fatal asthma is 25 to 40mg/kg/dose, infused intravenously, over 20 to 30 minutes.^(53)^ The onset of clinical response is rapid (occurring in minutes) and generally is observed during the initial infusion. Patients should be carefully monitored for adverse effects during the infusion, which include hypotension, nausea, and flushing. Serious toxicity, such as cardiac arrhythmias, muscle weakness, areflexia, and respiratory depression, can occur but rarely is of significant concern when the correct regimen is used. The IV infusion of magnesium sulfate under controlled conditions appears to be safe, and a subset of patients with critical and near-fatal asthma clearly responds to this therapy, which may reduce need for mechanical ventilation.^(14,51-54)^ A systematic review of the published randomized controlled trials supports the use of magnesium sulfate in addition to β_2_-agonist agents and systemic steroid drugs in the treatment of persons with acute severe asthma.^(55)^ We typically reserve IV magnesium for children who are progressing towards respiratory failure despite therapy with systemic corticosteroids, β-agonists and ipratropium bromide.

## METHYLXANTHINE AGENTS

Methylxanthine agents, such as theophylline and aminophylline, promote bronchodilation by inhibiting phosphodiesterase-4 and increasing levels of cAMP.^(56)^ Other mechanisms of action have been proposed, including adenosine receptor antagonism and release of endogenous catecholamines.^(57,58)^ Theophylline also has anti-inflammatory actions and is known to augment diaphragmatic contractility and increase respiratory drive.^(59,60)^ Side effects generally are seen at serum concentrations > 15-20µg/mL and include nausea, vomiting, dysrhythmia, dyskinesias, seizures, and death. The therapeutic range is 10 - 20µg/mL, so these drugs have a very narrow therapeutic window. Aminophylline is preferred over theophylline in the ICU because it is parenterally administered. A loading dose (we use 5.7mg/kg) typically is administered over 20 minutes and should be followed immediately by the continuous infusion of the drug. Starting infusion rates range from 0.5 - 1mg/kg/h and are age dependent. Lower doses should be used in the presence of compromised hepatic or cardiovascular function, and obese patients should have doses calculated based on ideal body weight to decrease the likelihood of toxicity. Serum drug levels should be monitored 30 to 60 minutes after the loading dose and frequently during the continuous infusion, considering that steady-state concentrations are not achieved until approximately five half-lives, which corresponds to 24 to 36 hours of infusion. Aminophylline and theophylline may lead to faster improvements in respiratory distress scores and pulmonary function testing but do not shorten ICU length of stay.^(61,62)^

Considering the very narrow therapeutic window, the questionable evidence of clinical efficacy, and the risk of severe side effects, use of these agents has decreased significantly. Methylxanthines were used in less than 6% of children with critical and near-fatal asthma admitted to pediatric ICUs in a recent multicenter study in the United States.^(63)^ We generally reserve aminophylline for selected patients who are progressing towards respiratory failure despite maximal therapy with systemic corticosteroids, β-agonists, ipratropium bromide, magnesium sulfate, and other adjuncts, while many intensivists have completely abandoned its use.

## HELIUM-OXYGEN MIXTURES

Helium is a biologically inert gas that is less dense than any other gas except hydrogen and is about one seventh as dense as air. Because of its low density, a mixture of helium and oxygen (heliox) reduces the Reynolds number and facilitates laminar gas flow in the airways, thus decreasing the work of breathing in situations associated with high airway resistance.^(64)^ To get the most benefit from the lower gas density, between 80:20 to 60:40 helium-oxygen mixtures must be used, limiting the therapy to those with low inspired oxygen needs. Because helium is inert, there are no side effects associated with its use other than potential hypoxemia. While the benefit of heliox is well established in children with extrathoracic airway obstruction, the role of heliox in patients with asthma is less clear.^(64,65)^ Heliox can improve pulmonary deposition of aerosolized drugs such as albuterol.^(66,67)^ Some data support that heliox-driven continuous nebulized albuterol treatments are associated with a greater degree of clinical improvement compared with oxygen-driven continuously nebulized albuterol in children with moderate to severe asthma exacerbations, but other studies have shown no significant improvement in hospital or ICU length of stay.^(66,68)^ Although some centers use heliox commonly, we rarely administer it to our patients with critical asthma.

## KETAMINE

Ketamine hydrochloride is a dissociative anesthetic agent with bronchodilatory properties via blockage of *N*-methyl-d-aspartate receptors in airway smooth muscle.^(69)^ Usual ketamine doses do not significantly affect hypoxic or hypercarbic respiratory drive. Pharyngeal and laryngeal reflexes are maintained, and although the cough reflex is somewhat depressed, airway obstruction does not normally occur. Case reports describe that ketamine may stave off endotracheal intubation in select patients, but ketamine infusion did not show clinical benefit in a randomized trial in the emergency department.^(70)^ In our experience, the administration of ketamine to non-intubated children with critical asthma frequently precedes the need to intubate and is rarely associated with significant and noticeable clinical improvement. For this reason, attempts at administering ketamine to non-intubated children with severe critical asthma should always occur under strictly monitored conditions and with personnel capable of rapidly establishing an airway for initiation of mechanical ventilatory support. The bronchodilatory effect of ketamine makes it an attractive agent in patients with asthma who require sedation and anesthesia for intubation or mechanical ventilation.^(71,72)^ Ketamine usually is administered as an IV bolus of 1 - 2mg/kg, followed by a continuous infusion of 1 to 2mg/kg/h. Side effects include sialorrhea, which can be attenuated by glycopyrrolate or atropine, and hallucinations during emergence, which can be attenuated with benzodiazepines.^(73)^

## MECHANICAL VENTILATION

### Indications

Only a small minority of patients with critical asthma (10% to 12%) requires endotracheal intubation and mechanical ventilation.^(63)^ The indications for intubation are not precisely defined, and the decision to proceed with intubation is largely based on clinical judgment. Absolute indications are obvious and include cardiac or respiratory arrest. We institute mechanical ventilation in critical asthma patients who, despite maximal therapeutic efforts, have persistent hypoxemia, non-sustainable dyspnea, severe agitation, or obtundation. Some patients may benefit from attempts to attenuate respiratory muscle fatigue with a trial of noninvasive ventilation.^(74,75)^ However, the use of bi-level positive airway pressure (BiPAP) requires patient cooperation and a well-sealed mask, which may prove difficult, if not impossible, to achieve in an anxious and agitated child with impending respiratory failure. Sedation with low-dose ketamine or dexmedetomidine may facilitate tolerance of BiPAP but may also accelerate respiratory failure.

## INTUBATION, ANALGESIA, SEDATION, AND MUSCLE RELAXATION

Intubation of patients with near-fatal asthma is complicated by concurrent acidosis and hypoxemia, decreased venous return from positive airway pressure, and hemodynamic effects of medications used to facilitate intubation. The risk of peri-intubation cardiac arrest can be mitigated with pre-oxygenation, rapid intravenous fluid administration, thoughtful drug selection, prompt placement of a cuffed endotracheal tube, and avoidance of hyperventilation. In our practice, we use ketamine to provide anesthesia and a fast non-depolarizing neuromuscular antagonist (i.e., rocuronium). A benzodiazepine (i.e., midazolam) is commonly used as an adjunct to provide additional sedation and mitigate emergence reactions, but administration may be delayed until there is hemodynamic stability following successful intubation. Care must be exercised with other sedative agents, particularly propofol, in patients who may not tolerate the potential negative hemodynamic side effects. Once intubated, the patient must be hand-ventilated with an appropriately slow rate to permit complete exhalation and avoid hyperinflation, hypoxemia, and hemodynamic instability prior to connection to the mechanical ventilator. Tension pneumothorax should be considered if refractory hypoxemia and hypotension develop.

After intubation, ongoing analgesia and sedation are needed to avoid tachypnea, breath stacking, and ventilator dyssynchrony, particularly in the setting of permissive hypercapnia. We typically continue ketamine as an infusion (1 to 2mg/kg/h, IV). Its use with continuous infusions of midazolam (0.1 to 0.2mg/kg/h, IV) can provide deep sedation while decreasing the chance of hallucinatory reactions. For additional analgesia, we prefer fentanyl over morphine due to the latter's ability to promote histamine release. We continue neuromuscular blockade until satisfactory gas exchange and clinical stability are achieved, which often takes 1 or 2 days. Patients who exhibit significant hypercapnia during mechanical ventilation may require continuation of neuromuscular blockers to abolish spontaneous respiratory movements that could worsen dynamic hyperinflation. However, use of neuromuscular blockers should be discontinued as soon as feasible to reduce the likelihood of prolonged muscle weakness from the interaction of these agents and corticosteroids.^(76,77)^ We prefer to use cisatracurium because it does not contain the corticosteroid-like moiety found in vecuronium and rocuronium that is thought to explain the association between myopathy and co-therapy with both corticosteroids and aminosteroid-based neuromuscular antagonists.^(76,77)^

## VENTILATOR SETTINGS

**Figure 1:**
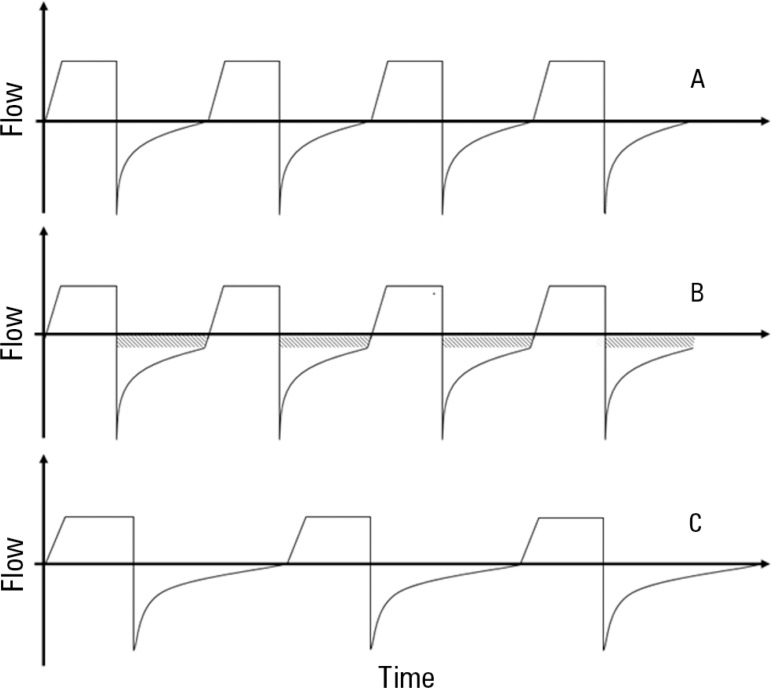
Schematic representation of the airway gas flow tracing over time during volume control ventilation. A) Normal tracing with no evidence of increased airway resistance. B) Expiratory flow does not return to zero prior to the initiation of the following breath, resulting in gas trapping and auto-PEEP. C) After ventilator setting optimization (lower respiratory rate and longer expiratory time), expiratory flow returns to baseline prior to initiation of the following breath.

The goal of mechanical ventilation in patients with near-fatal asthma is not to normalize the arterial blood gases but to reverse hypoxemia (if present), relieve respiratory muscle fatigue, and maintain a level of alveolar ventilation compatible with an acceptable pH, while avoiding iatrogenic hyperinflation and levels of intrathoracic pressure that reduce cardiac output. A strategy involving permissive hypercapnia and robust tidal volumes (8 - 12mL/kg) has been associated with very low mortality rates in adults and children with near-fatal asthma.^(78,79)^ Targeting a normal PaCO_2_ would be ill-advised, as this would require fast respiratory rates and high minute volumes that lead to hyperinflation and increase the risk of pneumothorax, pneumomediastinum, and death.^(78-80)^ Close attention should be given to chest auscultation and ventilator flow-time curves, as initiation of a new breath prior to cessation of expiratory flow from the previous breath will also lead to increased hyperinflation (Figure 1).

The most common modes of mechanical ventilation in children with near-fatal asthma are the synchronized forms of pressure control, volume control, pressure-regulated volume control (PRVC), and pressure support with positive end-expiratory pressure (PEEP).^(10)^ No definitive evidence exists to suggest that one mode of ventilation is superior to the other. We generally avoid pressure control due to wide variability in tidal volumes influenced by the ever-changing airway resistance to gas flow. Volume control regulates tidal volumes and allows for accurate comparisons of peak inspiratory and plateau pressures but may lead to excessive peak inspiratory pressures and breath stacking. PRVC similarly assures tidal volumes but provides decelerating flow (seen by many as advantageous in distal airway obstruction) and lower peak inspiratory pressures. Pressure support with PEEP may markedly improve ventilation by allowing the patient to control inspiratory times and rates, and enabling the patient to actively assist with exhalation.^(81)^ Some practitioners use pressure support with PEEP early after intubation, but it is more commonly used in patients who are nearing extubation.^(10)^

**Figure 2:**
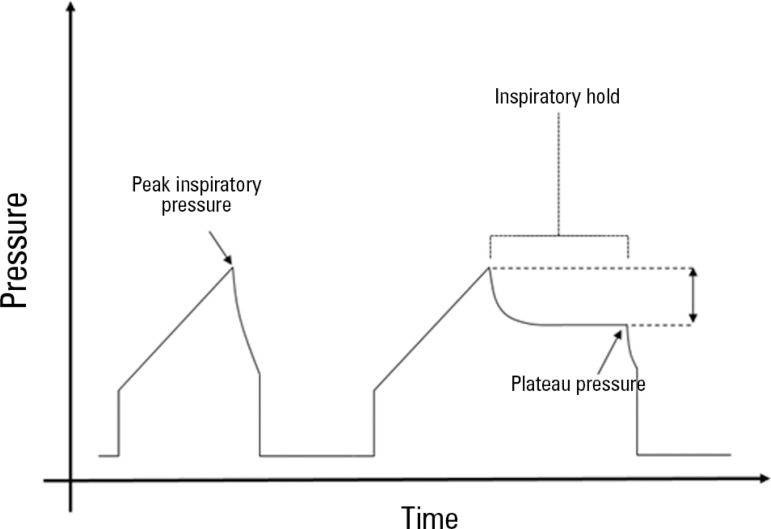
Schematic representation of the airway pressure waveform over time during volume control ventilation. The peak-to-plateau pressure difference (double-headed arrow) is obtained after an inspiratory hold by comparing the peak pressure and the measured plateau pressure.

**Figure 3:**
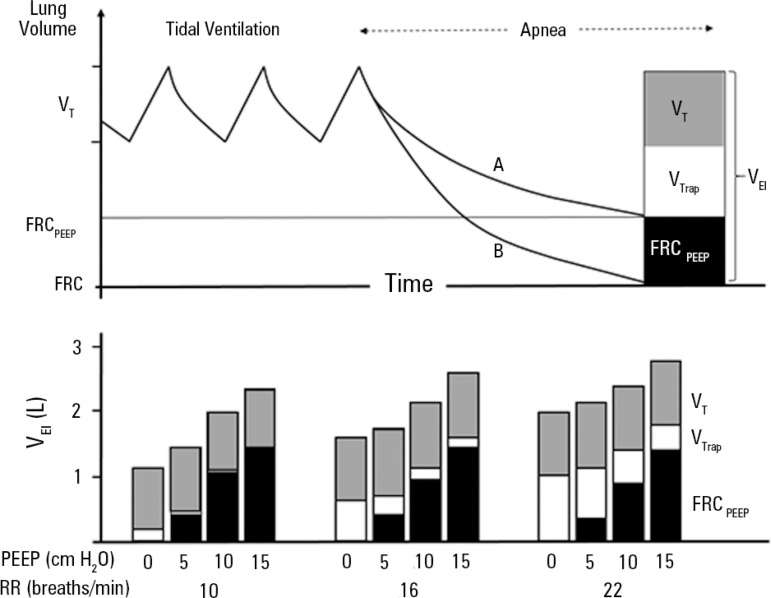
Upper panel: schematic representation of the measurement of end-inspiratory lung volume above functional residual capacity both with (A) and without (B) positive end-expiratory pressure by a period of apnea during steady-state ventilation. Lower panel: the effect of positive end-expiratory pressure (0, 5, 10 and 15cmH_2_O) on lung volumes at each level of minute ventilation (respiratory rate 10, 16 and 20 breaths/min). Note that the application of positive end-expiratory pressure leads to a progressive increase in lung volume due to increased functional residual capacity and volume of trapped gas above functional residual capacity, particularly at faster respiratory rates. FRC - functional residual capacity; FRC_PEEP_ - functional residual capacity resulting from PEEP; PEEP - positive end-expiratory pressure; V_EI_ - end-inspiratory lung volume above FRC; V_T_ - tidal volume; V_Trap_ - volume of trapped gas above FRC; RR - respiratory rate. Source: Tuxen DV. Detrimental effects of positive end-expiratory pressure during controlled mechanical ventilation of patients with severe airflow obstruction. Am Rev Respir Dis. 1989;140(1):5-9.^(82)^

Our preference is to initially use the volume control synchronized mandatory ventilation mode (VC-SIMV) or the pressure regulated volume control mode (PRVC), with tidal volumes of 8 to 12mL/kg, which can be reduced as needed to generate peak inspiratory pressures of 45cmH_2_O or less and plateau pressures of 30cmH_2_O or less. In cases with very severe airway obstruction, peak inspiratory pressures in excess of 50 or 60cmH_2_O might be generated, and it is imperative in these cases that the plateau pressure be frequently monitored and kept at a safe level of 30cmH_2_O or less. The plateau pressure (Figure 2) is measured during an inspiratory hold at the end of inspiration, after pressure equilibration and in the absence of gas flow - thus it is not affected by the degree of airway obstruction (unlike the peak inspiratory pressure). Although very high peak inspiratory pressures (measured dynamically during the inspiration) might indicate severe obstruction to airflow in the sickest patients under VC-SIMV, the alveoli are not directly exposed to these pressures but to the statically measured plateau pressures. Therefore, maintaining the plateau pressure ≤ 30cmH_2_O should lower the likelihood of pneumothorax and other ventilator-associated lung injury. The respiratory rate is initially set between 6 and 12 breaths/min, and inspiratory time is set between 1 and 1.5 seconds, allowing for expiratory times between 4 and 9 seconds. Younger patients may need somewhat higher rates, but the ratio of inspiratory time to expiratory time (I:E ratio) should always be set low. PEEP is set at zero for patients under neuromuscular blockade, as the application of any PEEP to such patients is associated with higher lung volumes (Figure 3), increased airway and intrathoracic pressures, and circulatory compromise.^(82)^

**Figure 4:**
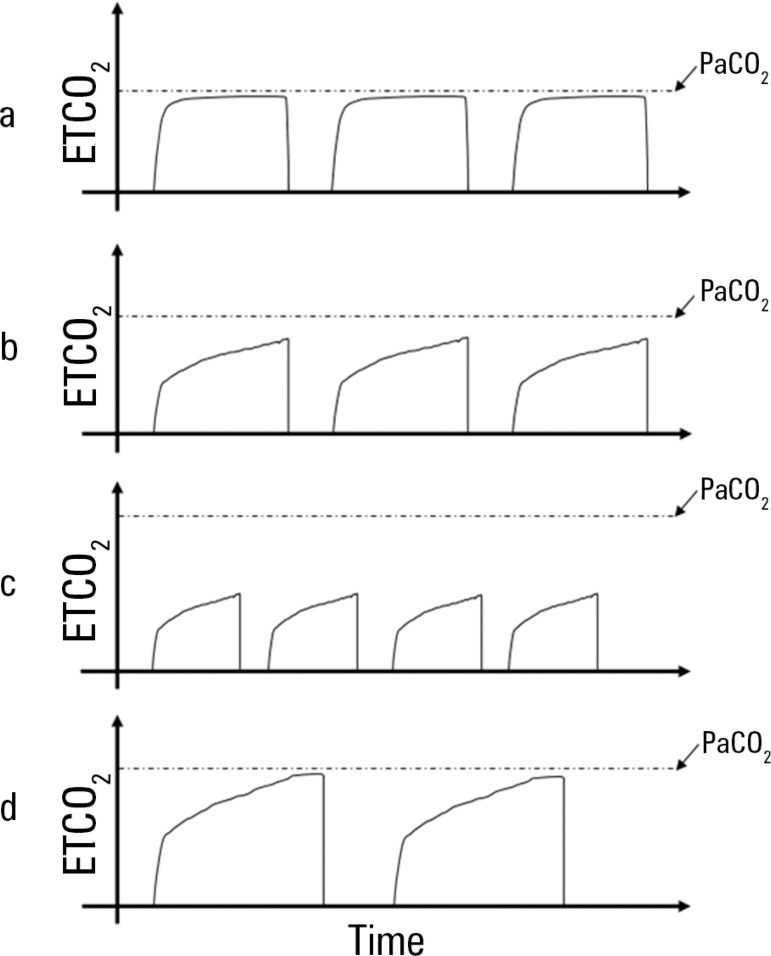
Schematic representation of capnogram tracings under various clinical conditions. The interrupted lines mark the reference value for arterial partial pressure of carbon dioxide. Under normal conditions (a), the end-tidal carbon dioxide tracing plateaus during exhalation and approximates the partial pressure of carbon dioxide. In near-fatal asthma (b) severe airflow obstruction is manifested by the up-sloping of the expiratory phase tracing and absence of a plateau, suggesting incomplete exhalation prior to the following inspiration. Note the wider gap between the end-tidal carbon dioxide and partial pressure of carbon dioxide. Attempting to address the higher partial pressure of carbon dioxide by increasing the respiratory rate (c) leads to an even higher partial pressure of carbon dioxide and a wider gap between the partial pressure of carbon dioxide and end-tidal carbon dioxide, along with hyperinflation and its attendant side effects. Decreasing the respiratory rate (d) leads to a longer expiratory time and more complete exhalation, with an end-tidal carbon dioxide measurement that more closely reflects the partial pressure of carbon dioxide. ETCO_2_ - end-tidal carbon dioxide; PaCO_2_ - partial pressure of carbon dioxide.

We do not target a specific pH or PaCO_2_ goal but rather attempt to optimize ventilation via frequent auscultation and analysis of ventilator waveforms and loops. The difference between peak inspiratory pressure and plateau pressure is directly related to resistance of the airways and can be monitored to assess response to treatment. This peak-to-plateau difference may be spuriously low if PRVC is employed due to the decelerating flow pattern and lower peak inspiratory pressures in this ventilator mode. A continuous upslope of the capnography curve ("ramping") indicates significant airways obstruction and can similarly be used as a measure of disease severity (Figure 4). When ramping is present and end-tidal CO_2_ does not reach steady state, shortening the expiratory time (i.e., increasing the respiratory rate) will worsen ventilation but decrease the end-tidal CO_2_ by truncating the exhalation earlier in the breath, giving the false impression that ventilation has improved. Frequent arterial blood gas measurements are needed to accurately assess ventilation in the acute stages. It is important to not reflexively increase the ventilator rate if excessive hypercarbia is present, as increasing the ventilator rate shortens time for exhalation and can further increase PaCO_2_.

With clinical improvement, the neuromuscular blockade should be stopped and trigger sensitivity for spontaneous breaths should be optimized. Once the patient no longer requires neuromuscular blockade, a low level of PEEP (lower than the measured auto-PEEP and generally not in excess of 8cmH_2_O) is applied to facilitate synchronization with the ventilator. In this setting, PEEP may improve lung mechanics by moving the equal pressure point further down the airways and enabling decompression of upstream alveoli and by facilitating ventilator triggering and synchronization.^(83,84)^ Patients often are liberated from mechanical ventilation while significant symptoms still persist, as long as gas exchange is stable and acceptable using pressure support with PEEP and the peak of bronchospasm has passed.

## REFRACTORY CASES

When oxygenation and ventilation are still inadequate despite mechanical ventilation, treatment options include bronchoscopy, inhalational anesthesia, and extracorporeal life support (ECLS).^(10,85-88)^ Bronchoscopy, which was employed during mechanical ventilation in 7% of the patients in the CPCCRN cohort, may remove the significant mucus plugs found in some patients with near-fatal asthma^(10,89-91)^ but may have little effect in patients where mucosal edema predominates and even aggravate bronchoconstriction. Inhalational agents such as isoflurane and sevoflurane are potent bronchodilators that have been used in children with near-fatal asthma.^(92,93)^ Their use is limited by technical issues and safety concerns. Most ventilators used in the ICU do not have a scavenger system for proper disposal of inhaled anesthetics, and anesthesia machines commonly used in the operating room may not be sufficiently sophisticated to ventilate children with near-fatal asthma. Side effects include hypotension, arrhythmias, and movement disorders.^(92,93)^ Inhaled anesthesia was reported in 3% of cases in the CPCCRN study, while ECLS was used in only 1%.^(10)^ As of 2015, there were 256 reported cases of ECLS for near-fatal asthma (adults and children).^(94)^ Interestingly, the survival rate in these cases is approximately 83%, which is remarkable considering that the vast majority of these patients were extraordinarily sick and had failed to respond to very aggressive treatment.^(94)^

## PROGNOSIS

The prognosis of patients with critical or near-fatal asthma who receive proper medical therapy is excellent. Better understanding of the pathophysiology of airway obstruction and dynamic hyperinflation, coupled with improved mechanical ventilation strategies and aggressive pharmacologic treatment, has reduced the ICU mortality rate to nearly zero in these patients.^(10,95-97)^ Currently, most deaths from asthma occur in patients who suffered pre-hospital cardiac arrest and are related to its attendant catastrophic neurologic consequences.^(10-12,63)^

The post-discharge treatment plan for patients admitted to the hospital with critical or near-fatal asthma should be carefully reviewed prior to discharge to ensure adequate outpatient therapy, education, and follow-up in an attempt to reduce the likelihood of a preventable recurrence. Such patients should be followed by an asthma expert in addition to a pediatrician.

### Author contributions

All authors contributed equally to the concept, development, draft, and review of this manuscript.
